# A randomized clinical trial of on-demand oral pre-exposure prophylaxis does not modulate lymphoid/myeloid HIV target cell density in the foreskin

**DOI:** 10.1097/QAD.0000000000003619

**Published:** 2023-06-06

**Authors:** Cosnet L. Rametse, Emily L. Webb, Carolina Herrera, Berenice Alinde, Asiphe Besethi, Bongani Motaung, Tshepiso Mbangiwa, Lloyd Leach, Shorok Sebaa, Azure-Dee A.P. Pillay, Thabiso B. Seiphetlo, Boitshoko Malhangu, Stefan Petkov, Laura Else, Susan Mugaba, Patricia Namubiru, Geoffrey Odoch, Daniel Opoka, Jennifer Serwanga, Andrew S. Ssemata, Pontiano Kaleebu, Saye Khoo, Limakatso Lebina, Neil Martinson, Francesca Chiodi, Julie Fox, Clive M. Gray

**Affiliations:** a Division of Immunology, Department of Pathology, University of Cape Town, South Africa; b Medical Research Council (MRC) International Statistics and Epidemiology Group, London School of Hygiene & Tropical Medicine; c Department of Infectious Disease, Imperial College London, London, UK; d Division of Molecular Biology and Human Genetics, Biomedical Research Institute, Stellenbosch University, Cape Town, South Africa; e Department of Microbiology, Tumor and Cell Biology, Karolinska Institutet, Stockholm, Sweden; f Department of Pharmacology, University of Liverpool, Liverpool, UK; g MRC/Uganda Virus Research Institute/London School of Hygiene & Tropical Medicine Uganda Research Unit, Entebbe, Uganda; h University of the Witwatersrand Perinatal HIV Research Unit, Johannesburg, South Africa; i King's College London, London, UK.

**Keywords:** claudin-1, emtricitabine–tenofovir alafenamide, emtricitabine–tenofovir disoproxil fumarate, foreskin, HIV target cells, pre-exposure prophylaxis, voluntary medical male circumcision

## Abstract

**Objectives:**

As topical pre-exposure prophylaxis (PrEP) has been shown to cause immune modulation in rectal or cervical tissue, our aim was to examine the impact of oral PrEP on lymphoid and myeloid changes in the foreskin in response to dosing and timing of drug administration.

**Design:**

HIV-negative male individuals (*n* = 144) were recruited in South Africa and Uganda into an open-label randomized controlled trial in a 1 : 1 : 1 : 1 : 1 : 1 : 1 : 1 : 1 ratio to control arm (with no PrEP) or one of eight arms receiving emtricitabine–tenofovir disoproxil fumarate (F/TDF) or emtricitabine–tenofovir alafenamide (F/TAF) at one of two different doses, 5 or 21 h before undergoing voluntary medical male circumcision (VMMC).

**Methods:**

After dorsal-slit circumcision, foreskin tissue sections were embedded into Optimal Cutting Temperature media and analysed, blinded to trial allocation, to determine numbers of CD4^+^CCR5^+^, CD1a^+^ cells and claudin-1 expression. Cell densities were correlated with tissue-bound drug metabolites and p24 production after ex-vivo foreskin challenge with HIV-1_bal_.

**Results:**

There was no significant difference in CD4^+^CCR5^+^ or CD1a^+^ cell numbers in foreskins between treatment arms compared with the control arm. Claudin-1 expression was 34% higher (*P* = 0.003) in foreskin tissue from participants receiving PrEP relative to controls, but was no longer statistically significant after controlling for multiple comparisons. There was neither correlation of CD4^+^CCR5^+^, CD1a^+^ cell numbers, or claudin-1 expression with tissue-bound drug metabolites, nor with p24 production after ex-vivo viral challenge.

**Conclusion:**

Oral doses and timing of on-demand PrEP and in-situ drug metabolite levels in tissue have no effect on numbers or anatomical location of lymphoid or myeloid HIV target cells in foreskin tissue.

## Introduction

Due to a combination of antiretroviral availability, increased HIV testing and introduction of a range of HIV prevention tools, HIV incidence and mortality in African has declined [1]. However, HIV remains a global burden, with approximately 320 000 people newly infected in Africa in 2022 [1]. The most common mode of transmission is by heterosexual intercourse with HIV incidence highest among women of reproductive age [2]. Most HIV prevention strategies have focused on preventing male-to-female HIV transmission, whereas preventing HIV acquisition in men is relatively understudied.

Voluntary medical male circumcision (VMMC) has been shown to reduce HIV infection in men by 50–60% [3–5], demonstrating that the foreskin plays an important role in HIV susceptibility in male individuals. As a result, VMMC has been rolled out as a standard of preventive care in Africa, with more than 26 million circumcisions having been performed between 2008 and 2019. Although effective, VMMC uptake is unevenly distributed across countries in Africa [6], and additional prevention strategies are required, including pre-exposure prophylaxis (PrEP). On-demand oral doses of tenofovir (TDF/FTC) PrEP has been shown to be highly effective in MSM and heterosexual HIV-serodiscordant couples [7–9]. Furthermore, on demand emtricitabine–tenofovir disoproxil fumarate (F/TDF) and daily emtricitabine–tenofovir alafenamide (F/TAF) are also highly effective in MSM [10]. Tenofovir gel has been evaluated for vaginal and rectal PrEP [11,12], with the CAPRISA 004 study [12] showing up to 54% lower HIV incidence in women in the gel arm. However, women receiving 1% tenofovir gel had an accumulation of activated endocervical CD4^+^ T cells, which were associated with increased drug metabolite levels in the tissue [13]. Although these studies focused on topically applied PrEP, their findings raise the issue of host immune activation in response to drug, which may undermine the potential efficacy of PrEP. In an effort to identify the impact of oral PrEP in young men on immune status, we undertook a randomized clinical trial [14] to test the secondary outcome of immunological safety by measuring numbers of CD4^+^CCR5^+^ and CD1a^+^ cells in relation to claudin-1 barrier integrity in the foreskin.

## Methods

### Study design and participants

Between 19 October 2019 and 5 March 2021, 144 HIV-negative males aged 13–24 years were enrolled from South Africa and Uganda (72 per country) into an open-label controlled trial (NCT03986970) and randomized in a 1 : 1 : 1 : 1 : 1 : 1 : 1 : 1 : 1 ratio to control arm (no PrEP) or one of eight arms receiving F/TDF or F/TAF at one of two different doses (double dose on day 1 only, or double dose on day 1 plus single dose on day 2) before undergoing a standard of care programme of VMMC at 5 or 21 hours after receiving drug [14]. Participants from communities in South Africa and Uganda expressing an in interest in VMMC were approached to participate in the clinical trial. Participants took tablets at each clinic site under directly observed therapy (DOT). In Uganda, participants stayed overnight in the hospital for the 21-h dose but in SA they went home and returned the next day. Eligibility criteria included being clinically eligible for circumcision, weighing >35 kg, and being able to give written informed consent. Supplementary Figure 1 shows the number of participants screened and the numbers enrolled into each arm that went onto be circumcised.

### Randomization and masking

Random allocation sequence was generated by an independent statistician using Stata, stratified by country and using a block size of 9. Participants not able to continue in the study following randomization were excluded according to protocol, and an additional set of randomization codes generated using the same approach, to ensure a target sample size of 16 evaluable participants per trial arm was attained. Sequentially numbered opaque envelopes labelled with unique randomization identifier and containing the allocated intervention arm were prepared by two administrators not otherwise involved in the study. At time of randomization, clinical staff opened the sequential envelope and scheduled the participant to receive PrEP (if applicable) and VMMC, as per randomization arm. Participants and care providers were not blinded to trial arm. Laboratory outcome assessors were blinded until all measurements were completed. An independent statistician scrambled sample identifiers for laboratory investigation using Stata.

### Tissue sampling

VMMC was performed using the dorsal slit method. Foreskin tissue was placed immediately in cold DMEM and transported to the laboratory on ice within 30 min. All tissues were immediately dissected into 8 × 2.5 mm^2^ pieces (four for inner and four for outer) and snap-frozen in Optimal Cutting Temperature (OCT) compound and stored in −80°C freezers and were all later shipped to the University of Cape Town and processed for immunohistochemistry, imaging and analysis as previously described [15].

### Immunohistochemistry imaging and analysis

The density of CD4^+^CCR5^+^ cells was assessed from 72 participants from South Africa and 40 from Uganda. A more limited number of samples were assessed for CD1a and claudin-1 expression including all control arm participants (*n* = 16) and four randomly selected from each of the eight treatment trial arms (*n* = 40 from South Africa and 40 from Uganda = 80 in total). Supplementary Figure 1 shows the breakdown of participants analysed per arm along with the number of images collected and quantified. OCT snap-frozen tissues were sectioned (5–10 μm thick) using a Leica CM1850 Cryostat (Illinois, USA) and processed as described [15]. Tissues was stained with primary antihuman CD4^+^ antibody (1 : 50 dilution; Sigma, St. Louis, Missouri, USA); secondary Cy5 antibody (1 : 500; AEC Amersham, Johannesburg, South Africa); antihuman primary MC-5 CCR5 antibody (1 : 10; courtesy of Prof. Mathias Mack) and secondary Cy3 antibody (1 : 1000; AEC Amersham); primary antihuman claudin-1 antibody (1 : 200; Thermofisher, Waltham, Massachusetts, USA) and secondary DarB antibody (1 : 1000, AEC Amersham); primary antihuman CD1a antibody (Ltc Tech SA, Randburg, South Africa) and secondary Cy5 antibody (1 : 500; AEC Amersham). Cell nuclei were stained with DAPI (Hoescht). Negative controls were stained with isotype mouse or rabbit and kidney tubular tissue and positive controls for CCR5 on tonsular tissue (Supplementary Figure 2). Ten images for each section were collected using DeltaVision RT systems and softWoRx software (Applied Precision Instruments) and quantified using Image J and Pipsqueak (Supplementary Figure 3). Percentage of claudin-1 expression was calculated by dividing the number of pixels taken up by claudin-1 staining (Fig. 2d and e) by the total epidermal area for each foreskin section. We also delineated between CD1a^+^ cells inside the claudin-1 staining (intrinsic, iCD1a) and CD1a^+^ cells outside of the claudin-1 stain (extrinsic, eCD1a) and expressed this as a ratio: eCD1a/iCD1a.

### Ex-vivo challenge of foreskin tissue

Foreskin tissue was cut into 2 mm^2^ explants [16] and cultured by adding an outer and an inner explant per well, in DMEM supplemented with 10% FBS, 2 mmol/l l-glutamine, 2.5 μg of amphotericin B/ml, and antibiotics (100U of penicillin/ml, 100 μg of streptomycin/ml) (Sigma). Explants were immediately challenged with HIV-1_BaL_ at either a high titre [10^4^ TCID_50_/ml (median tissue culture infective dose/mL)] or low titre (2 × 10^2^ TCID_50_/ml), being more physiologically relevant. Control explant tissues received no virus. Foreskin explants were cultured for 15 days with approximately two-thirds of culture supernatant harvested at days 3, 7, 11 and 15, and cultures replenished with fresh medium. p24 was measured in the supernates by ELISA (Innotest HIV antigen mAb ELISA, Fujirebio Europe, Belgium). The lower limit of quantification (LLQ) for the assay was 0.02998 and p24 concentrations that were below the LLQ of each assay were expressed as half-LLQ values.

### Tissue drug metabolites

Concentrations of the active phosphorylated intracellular metabolites – tenofovir-diphosphate (TFV-DP) and emtricitabine-triphosphate (FTC-TP) were determined in foreskin tissue as previously described [17].

### RNA sequencing of foreskin tissue

The details of the procedure used for RNA sequencing of foreskin tissue have been previously described [18]. Transcriptomes were derived from combined inner and outer foreskin from each participant.

### Statistical analysis

The following outcomes were summarized both overall and by trial arm: CD4^+^ cell density, CCR5 density, CD4^+^CCR5^+^, proportion of CCR5 expression on CD4^+^ cells, distance from the epidermis, eCD1a/iCD1a ratio, CD1a/cm^2^, % claudin expression. Except for the proportion of CCR5 expression on CD4^+^ cells, outcomes were positively skewed and were, therefore, log-transformed for analysis. The relative effect of trial interventions was assessed through the following comparisons: any PrEP versus control arm, F/TAF versus F/TDF, 2+1 tablets versus 2 tablets, 21 h between PrEP and VMMC versus 5 h. Further comparisons also assessed the effect of dosage separately for each drug, and the effect of the interval, separately for each drug and dosage. Mixed effects linear regression models, allowing for clustering within participants since several sections of tissue were imaged for each participant, were used to determine the mean difference and 95% confidence interval (CI) for each comparison, with *P* values determined by likelihood ratio tests. For all outcomes other than the proportion of CCR5 expression on CD4^+^ cells, parameters were back transformed and reported as geometric mean ratios (GMR). Correlations between each study outcome and pharmacokinetic–pharmacodynamic (PK–PD) parameters were assessed by Pearson's correlation coefficient, using the mean of the imaging outcome from the different sections for each participant.

### Ethics approvals

Written informed consent was obtained from all participants aged at least 18 years and emancipated minors (in Uganda); for those less than 18 years and not emancipated minors, their assent with parental consent was obtained. The trial was conducted in accordance with the principles of the Declaration of Helsinki and Good Clinical Practice and approved in the South African Health Products Regulatory Authority (20181004). Ethical approval was granted from University of Cape Town (290/2018), University of the Witwatersrand (180906B, M1811148 and 180108), Uganda Virus Research Institute research ethics committee (GC/127/18/12/680), Uganda National Council of Science and Technology (HS2534), Uganda National Drug Authority (618/NDA/DPS/09/2019) and London School of Hygiene and Tropical Medicine research ethics committee (17403). The Swedish Ethics Review Authority approved the laboratory studies of the collected specimens at the Karolinska Institute (2020-00941).

## Results

### Impact of emtricitabine–tenofovir disoproxil fumarate or emtricitabine–tenofovir alafenamide on the density of CD4^+^CCR5^+^ and CCR5 expression on CD4^+^ cells in the foreskin

**Fig. 1:**
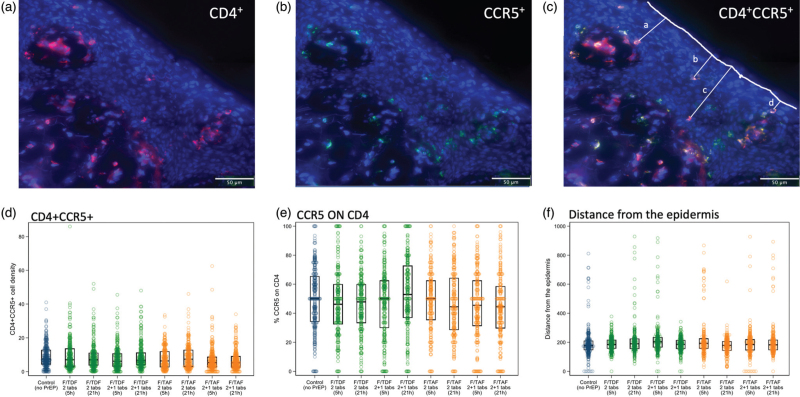
Density of CD4^+^CCR5^+^ and CCR5 on CD4^+^ cells in foreskins from the participants of the CHAPS trial.

As CCR5 is the main HIV co-receptor for most transmitted isolates [19,20], we assessed the density of double-expressing CD4^+^CCR5^+^ cells along with the proportion of CCR5 staining on CD4^+^ cells. Figure 1a–c show representative images of single-expressing (a and b) and double-expressing CD4^+^CCR5^+^ cells (c) in foreskin tissue in relation to the outer epidermis. Figure 1d shows the distribution of double-expressing CD4^+^CCR5^+^ cell density by treatment arm, where no significant differences were identified. Table 1 shows results from a mixed effects model where no difference was identified for any comparison between reference and comparator group. Additionally, timing and dose of F/TDF and F/TAF had no significant impact on CCR5 expression on CD4^+^ cells (Fig. 1e, Supplementary Table 1). This finding was consistent at the tissue gene expression level, where there was no significant difference in the expression of *CD4* or *CCR5* genes between treatment and control arms (Supplementary Figures 4A and B). Collectively, these data show that the density of HIV target cells and CCR5 expression was not modulated by drug dose or timing of PrEP prior to VMMC.

(a) Representative image of CD4^+^ cells in the foreskin; (b) representative image of CCR5^+^ cells in the foreskin; (c) representative image of dual stained CD4^+^CCR5^+^ cells in the foreskin showing the outline of the epidermis layer and representative distances (a, b, c and d) of cells from the surface; (d) numbers of CD4^+^CCR5^+^ cells in the foreskin across the nine trial arms. Box plots show median lines and interquartile ranges overlayed with each measurement. (e) Proportion of CCR5 expression on CD4^+^ cells in the foreskin across the nine trial arms. Box plots show median lines and interquartile ranges overlayed with each measurement. (f) Distance of CD4^+^CCR5^+^ cells within the foreskin from the outer epidermis from participants across the nine trial arms. Box plots show median lines and interquartile ranges overlayed with each measurement.

**Table 1:** Comparisons between trial arms for CD4^+^CCR5^+^, CD1a^+^ cells, claudin-1 expression and ratio of eCD1a/iCD1a.

		CD4^+^CCR5^+^/cm^2^		CD1a^+^/cm^2^		% claudin-1 expression		ratio eCD1a/iCD1a	
Reference group	Comparator group	Geometric mean ratio (95% CI)	*P*	Geometric mean ratio (95% CI)	*P*	Geometric mean ratio (95% CI)	*P*	Geometric mean ratio (95% CI)	*P*
Effect of any PrEP versus no PrEP									
Control arm	Any PrEP	0.84 (0.50–1.41)	0.51	1.10 (0.89–1.35)	0.39	**1.34 (1.11–1.62)**	**0.003**	0.92 (0.75–1.13)	0.43
Overall effects of drug, dosage and interval									
F/TDF (all)	F/TAF (all)	0.82 (0.55–1.22)	0.33	1.04 (0.86–1.26)	0.71	0.93 (0.79–1.10)	0.42	1.15 (0.95–1.39)	0.15
2 tabs (both drugs)	2+1 tabs (both drugs)	0.80 (0.54–1.19)	0.28	0.97 (0.80–1.18)	0.75	0.99 (0.83–1.17)	0.87	1.04 (0.85–1.26)	0.72
5 h (all regimens)	21 h (all regimens)	1.09 (0.74–1.63)	0.66	0.95 (0.78–1.15)	0.61	0.88 (0.75–1.04)	0.14	1.03 (0.85–1.25)	0.77
Effects of dosage, separately for each drug									
F/TDF, 2 tabs	F/TDF, 2+1 tabs	0.92 (0.55–1.53)	0.75	0.85 (0.65–1.11)	0.23	0.93 (0.73–1.19)	0.58	1.17 (0.88–1.56)	0.28
F/TAF, 2 tabs	F/TAF, 2+1 tabs	0.71 (0.38–1.30)	0.26	1.10 (0.83–1.45)	0.51	1.04 (0.81–1.32)	0.77	0.93 (0.72–1.19)	0.55
Effects of interval, separately for each drug and dosage									
F/TDF, 2 tabs (5h)	F/TDF, 2 tabs (21 h)	0.82 (0.40–1.68)	0.60	1.01 (0.69–1.47)	0.96	1.04 (0.76–1.42)	0.82	0.77 (0.50–1.17)	0.22
F/TDF, 2+1 tabs (5h)	F/TDF, 2+1 tabs (5 h)	1.35 (0.64–2.86)	0.43	0.81 (0.55–1.19)	0.28	0.83 (0.58–1.20)	0.33	1.39 (0.98–1.98)	0.07
F/TAF, 2 tabs (5h)	F/TAF, 2 tabs (21 h)	1.20 (0.52–2.75)	0.67	1.27 (0.91–1.76)	0.15	1.15 (0.91–1.47)	0.24	0.84 (0.58–1.22)	0.35
F/TAF, 2+1 tabs (5h)	F/TAF, 2+1 tabs (21 h)	1.07 (0.43–2.69)	0.88	0.80 (0.52–1.23)	0.31	**0.62 (0.43–0.88)**	**0.007**	1.24 (0.88–1.75)	0.21

Results from mixed effects models (each image measurement is included in the model and clustering of measurements within each participant is accounted for by including a random effect). CI, confidence interval; F/TAF, emtricitabine–tenofovir alafenamide; F/TDF, emtricitabine–tenofovir disoproxil fumarate; PrEP, pre-exposure prophylaxis. Bolded numbers represent significant values.

On a subset of samples (South Africa participants), we compared the impact of the drug dose and timing on the density of CD4^+^CCR5^+^ cells separately for inner and outer foreskin samples. Supplementary Figures 4A and B show that PrEP doses from the different arms had no impact on the density of CD4^+^CCCR5^+^ cells in either the inner or outer foreskin. There was also no significant difference (*P* = 0.14) in the densities of CD4^+^CCR5^+^ cells between the inner and outer foreskin. However, there was 5.8 times greater expression of CCR5 on CD4^+^ cells in the outer foreskin (*P* = 0.01), inferring that the outer foreskin would be more susceptible to HIV-1 infection.

We then tested the hypothesis that drug may influence the anatomical location of CD4^+^CCR5^+^ cells and possibly drive them either deeper into the tissue or further to the apical layer of the epidermis. Figure 1f and Supplementary Table 1 shows that the distance of potential HIV-1 target cells from the epidermis was not significantly different between treatment arms or relative to the control arm.

### Impact of emtricitabine–tenofovir disoproxil fumarate or emtricitabine–tenofovir alafenamide on the density of CD1a^+^ cells and claudin-1 expression in the foreskin

**Fig. 2:**
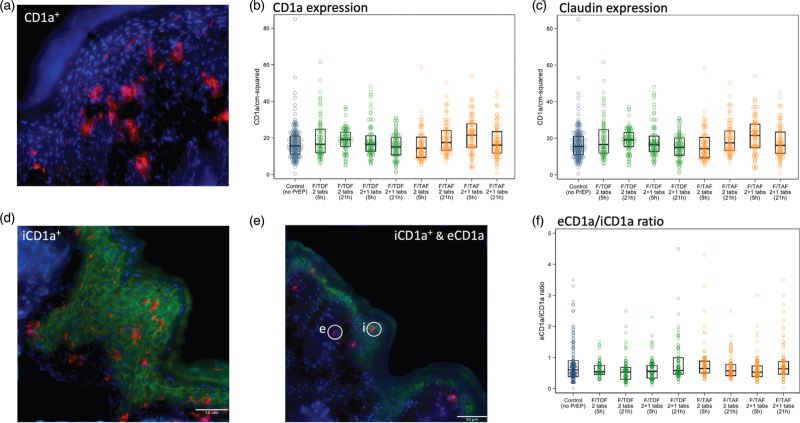
Density of CD1a^+^ and claudin-1 expression in the foreskins of men in different trial arms.

CD1a is one of the unique proteins that is expressed on either dendritic cells [21] or epidermal Langerhans cells [22,23]. Furthermore, this marker represents an important cell type as either an HIV target [24] or for delivery of HIV away from sites of infection [24]. We sought to identify whether either of the PrEP regimens, doses, or time of administration prior to VMMC impacted upon the density and location of CD1a^+^ cells from the outer epithelial layer, as measured by claudin-1, one of the tight junction proteins found in the foreskin epidermis [25,26]. Figure 2a shows a representative image of CD1a^+^ cell staining, illustrating the presence of these cells in both epidermis and dermis. Figure 2b and Table 1 shows that no significant differences existed when comparing between treatment arm permutations for combined samples from both South Africa and Uganda. This was also the case when we analysed separately by clinical site (South Africa versus Uganda, Supplementary Figure 6A and B), although there was a significantly lower density of CD1a^+^ cells in samples from Uganda (*P* = 0.03) compared with South Africa.

(a) Representative image of CD1a^+^ cells located in the foreskin; (b) combined density of CD1a^+^ cells in foreskins (cells/cm^2^) across the different trial arms; (c) combined expression of claudin-1 across the different trial arms; (d) representative image showing the presence of CD1a cells within (intrinsic, i) claudin-1 expression; (e) representative image of CD1a^+^ cells within (intrinsic, i) claudin-1 expression and outside (extrinsic, e). The white circled cells delineate iCD1a from eCD1a; (f) combined ratio of eCD1a/iCD1a across the trial arms.

In addition to using claudin-1 to delineate the outer epithelial surface, we quantified expression as a measure of barrier integrity [27]. Table 1 shows the geometric mean of percentage claudin-1 expression was 34% higher (*P* = 0.003) in combined inner and outer foreskin tissue from all participants receiving PrEP, compared with the control arm. After allowing for multiple comparisons, this was no longer significant (*P* = 0.288). Comparing F/TAF double dose given 5 h before VMMC versus F/TAF double dose given 21 h prior to circumcision, resulted in 28% lower expression of claudin-1 (*P* = 0.007, Table 1 and Fig. 2c), which was no longer significant after allowing for multiple comparisons.

### Impact of emtricitabine–tenofovir disoproxil fumarate or emtricitabine–tenofovir alafenamide on the anatomical location of CD1a^+^ cells in the foreskin

To better gauge the location of CD1a^+^ cells in relation to the epidermis, we measured the ratio of CD1a^+^ cells between those located within the area of claudin-1 staining (intrinsic, i), being exclusively expressed in the epidermis, or located outside claudin-1 expression (extrinsic, e). Figure 2d and e show representative images of iCD1a (d) and a mix of iCD1a and eCD1a staining (e), respectively. Figure 2f and Table 1 show that there were no differences in the anatomical location of CD1a^+^ cells between the eight treatment arms compared with the control arm, suggesting that drug exposure was not eliciting any migration of CD1a^+^ cells within the foreskin.

### No association between the density of CD4^+^CCR5^+^ and CD1a^+^ cells and claudin-1 expression with foreskin tissue drug metabolite levels or p24 production after ex-vivo viral challenge

**Fig. 3:**
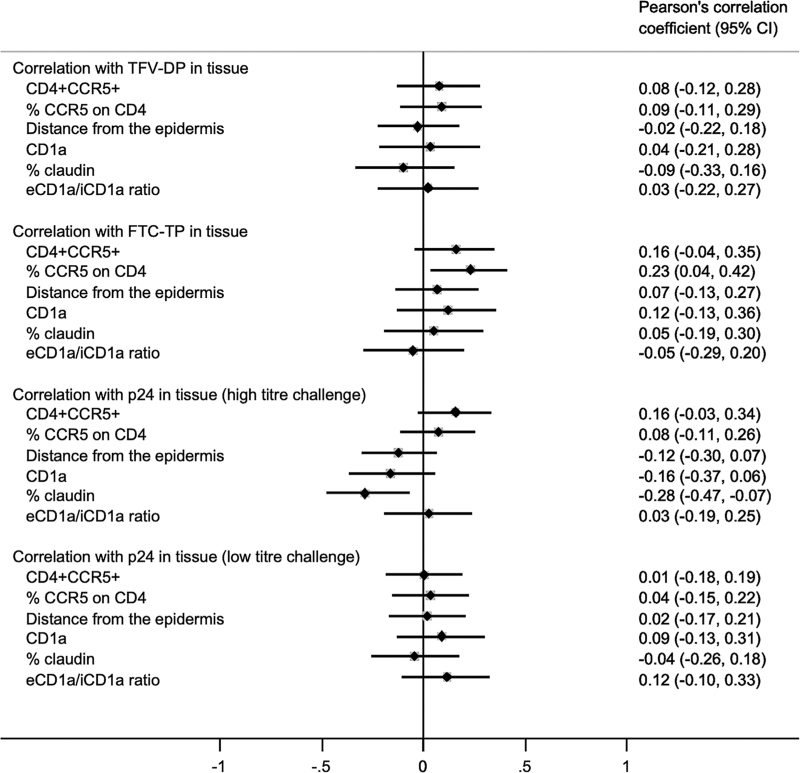
Forest plots of correlations of TVF-DP, FTC-TP and p24 (high and low dose) with log_10_ cell densities, distance from the epidermis, % claudin and eCD1a/iCD1a ratio.

One of the secondary objectives of the clinical trial was to identify the immune safety profile of the different PrEP dosing and timing [14]. Additional evidence to show this was made by correlating the density of HIV target cells with drug metabolite levels in the tissue. Figure 3 shows a lack of correlation between drug metabolites (TFV-DP and FTC-TP) in whole foreskin tissue with densities of CD4^+^CCR5^+^, CD1a^+^ cells, % CCR5 expression on CD4^+^ cells and claudin-1 expression. This would suggest that the protective effect of PrEP is independent of the density of HIV target cells in the tissue. This was underscored by a lack of association between CD4^+^CCR5^+^, % CCR5 expression and CD1a^+^ cell numbers with p24 production after ex-vivo challenge with low or high viral titre (Fig. 3). Likewise, there was no relationship between p24 production and the location of CD1a^+^ cells in relation to claudin-1 (Table 1). There was a weak negative association between claudin-1 expression and p24 production after high titre viral challenge (*r* = −0.28, *P* = 0.01, Fig. 3). This would suggest a trend of higher claudin-1 expression and an association with lower viral replication upon ex-vivo high-dose challenge.

Pearson correlation coefficients with 95% confidence intervals are shown for each comparison. The vertical line indicates a correlation coefficient of zero.

## Discussion

We show, in a randomized clinical trial [14], that short-course oral dosing and scheduling of on-demand F/TAF or F/TDF PrEP had no impact on the density of HIV target cells in foreskin tissue when men were circumcised at 5 or 21 h after taking drug. This was a novel trial where we aimed to show, as a secondary objective, that different types of PrEP and dosing schedules were not associated with immune activation *in situ.* We showed that CD4^+^CCR5^+^ cells, as measured quantitively and by gene expression levels in the tissue, were similar across different dosing and timing of drug administration. Importantly, we show that the expression of the HIV co-receptor CCR5 [18,28], well established to be increased upon cell activation [28,29], was not impacted by drug dose. This was similarly the finding with CD1a^+^ cells in the foreskin showing that epithelial dendritic cells [21,30] and/or epidermal Langerhans cells [23] were consistently present across the different trial arms but unchanged in numbers. In contrast to studies using topical PrEP whereby 1% tenofovir gel increased T-cell densities in rectal tissue [11] and CD4^+^ T-cell activation in endocervical tissue [13], we show in our study that oral PrEP dosing has no effect on local foreskin T-cell immunity. Previously, we showed that the CHAPS study resulted in modulation of gene expression in the foreskin resulting in a potentially unfavourable environment for HIV replication [18]. Collectively, these findings show that short-term oral PrEP does not induce immunologic activity in the foreskin.

The foreskin protects the host from invasion of a myriad of pathogens and does so by employing physical barriers and an intricate network of resident immune cells [31] and can be regarded as a persistently ‘inflamed’ tissue [32]. There is a fine distinction between Langerhans cells and dermal dendritic cells. Langerhans cells have been shown to reside in the epidermis and can act as immune sentinels by actively sampling environmental antigens [33], whereas dermal dendritic cells have been found in the papillary dermis [34]. CD1a has been shown to mark both dendritic cells [21] and epidermal Langerhans cells [22,23], and the epidermal location of CD1a^+^ cells in our study would be consistent with staining for Langerhans cells. Although we could not differentiate between these two myeloid populations, their numbers were not modulated by PrEP dosage or timing and nor associated with drug metabolites in the tissue or p24 production after low-dose or high-dose ex-vivo HIV challenge. Although the HIV challenge model is a proxy for in-vivo HIV transmission and acquisition in tissue, our finding suggests that the presence of in-situ HIV targets cells in the tissue is independent of downstream viral replication.

Physical barrier function in the skin resides within the stratum corneum, which relies on the arrangement of epithelial cells and tight junction proteins. The importance of tight junction proteins, claudin-1 particularly, was demonstrated to be crucial for survival in mice [27], where deletion of claudin-1 resulted in death shortly after birth because of defects in epidermal barrier function. There was tantalizing evidence, albeit not significant after multiple comparisons, that claudin-1 expression may be enhanced with PrEP. Interestingly, the shorter exposure to drug (5 h) resulted in lower claudin-1 expression compared with the longer duration (21 h) and infers that longer PrEP exposure may be more beneficial. This would suggest that barrier function may be enhanced with longer drug exposure and may represent a novel finding but needs to be explored in larger studies. It is known that respiratory epithelial barriers can be altered by type 2 cytokines (such as IL-4 and IL-13) and impacting on claudin and filaggrin to compromise epithelial barriers [35,36]. However, our observation that oral PrEP may enhance claudin-1 expression requires further validation experiments and offers a potentially unique insight into how PrEP may function beyond its direct anti-viral effects.

To our knowledge, this is the first time that the effects of oral PrEP has been assessed on immune cells in foreskin tissue. One of the limitations of this study was not being able to assess the impact of PrEP on separated inner and outer foreskin for all samples (Uganda, for example) because of inadequate sample collection. Additionally, this study does not address the possible role of fibroblasts and epithelial cells in the foreskin accumulating F/TAF and F/TDF [37] and the consequent long-term impact on numbers of CD4^+^CCR5^+^ and CD1a^+^ cells within the tissue, it does show that short-term ‘on-demand’ oral PrEP is immunologically well tolerated and does not induce higher numbers of activated CD4^+^ T cells in a vulnerable anatomical site for HIV acquisition in male individuals.

## Acknowledgements

We would like to thank all participants for their involvement in the study. Thanks to Matthias Mack for a generous donation of the anti-CCR5 antibody used in this study.

Author's contributions: C.H., J.S., P.K., S.K., N.M., F.C., J.F. and C.M.G. conceived and designed the study. C.L.R., E.L.W., S.M., L.W., P.N., G.O., D.O., A.S.S. and L.L. contributed to the implementation of the study in South Africa and Uganda. C.L.R., B.A., A.B., B.M., T.M., L.L., S.S., S.P., S.M., P.N., G.O., D.O., A.A.P.P. and T.B.S. contributed to the laboratory investigations and validation of the methods. C.L.R. and C.M.G. took the lead in writing the original draft and E.L.W. conceptualized and performed all statistical tests used in the article. All authors were involved in the review and editing of the manuscript. All authors had full access to all the study data and the final responsibility for the decision to submit for publication.

Financial support: this work was supported by the European and Developing Countries Clinical Trials 2 (EDCTP 2) programme of the European Union (RIA2016MC-CHAPS, awarded to J.F.) and the Swedish Research Council (2019–04596 Vetenskapsrådet awarded to F.C.). C.L.R. was supported by Clinician Researchers Programme award by the South African Medical Research Council (SAMRC) through its Division of Research Capacity Development. The content hereof is the sole responsibility of the authors and does not necessarily represent the official views of the SAMRC.

### Conflicts of interest

There are no conflicts of interest.

## Supplementary Material

Supplemental Digital Content

## Supplementary Material

Supplemental Digital Content

## Supplementary Material

Supplemental Digital Content
