# Supplementary material for: A randomized clinical trial of on-demand oral pre-exposure prophylaxis does not modulate lymphoid/myeloid HIV target cell density in the foreskin
Source: AIDS. 2023 Jun 6;37(11):1651–9. doi: 10.1097/QAD.0000000000003619 (PMC11175721; doi:10.1097/QAD.0000000000003619)
Supplement: Supplemental Digital Content [file aids-37-1651-s001.docx]

**Supplementary Figure 1:** Consort flow chart showing the number of participants screened and how many were analysed in each of the 8 treatment arms versus the control arm (no PrEP).

**Supplementary Figure 2. CCR5 positive and negative controls stainings:** A) Shows tonsillar tissue stained by with CCR5 antibody (green) and B) illustrated CCR5 staining of human renal tissue, which did not stain with the CCR5 antibody, both show nuclei staining in blue.

C

A

B

**Supplementary Figure 3. Quantification of immune cells in foreskin tissue. (**A) Representative image showing an ImageJ split image by channels; (B) automated quantification of CD4 cells on grayscale using Pipsqueak plug-in to ImageJ; (C) quantified CD4 cells superimposed on the original image showing how many are double-expressing CD4 and CCR5 or single expressing.

**Supplementary Figure 4. Gene expression of CD4, CCR5 and CD1a across the different trial arms.** Transcriptomic analysis of specific genes in foreskin tissue as assessed for (A) CD4, (B) CCR5 and (C) CD1a. Difference in normalised gene expression was tested between trial arms using analysis of variance as well as paired t-tests for each arms versus the control arm (no PrEP).

**Supplementary Figure 5. Density of CD4+CCR5+ in the inner and outer foreskins (FS)** (A) Combined density of CD4+CCR5+ cells in FS across the different trial arms in the inner FS from the SA site; (B) Combined density of CD4+CCR5+ cells in foreskins across the different trial arms in the outer FS from the SA site. Comparisons between inner and outer samples used paired t-tests to assess whether outcomes were the same in inner versus outer tissue samples.

p=0.03

**Supplementary Figure 6: Density of CD1a and claudin 1 expression in foreskins across treatment arms from two CHAPS sites in Africa.** (A) Density of CD1a (cells/cm^2^) in foreskins across treatment arms from South Africa participants (n=40); (B) Density of CD1a (cells/cm^2^) in foreskins across treatment arms from Uganda participants (n=40); (C) Percent expression of claudin-1 in foreskin tissue across treatment arms from South Africa participants (n=40); (D) Percent expression of claudin-1 in foreskin tissue across treatment arms from Uganda participants (n=40).

**Supplementary Figure 7: Ratio of eCD1a/iCD1a Langerhan’s cells in the foreskin (FS) from South Africa and Uganda participants.** (A) Ratio of CD1a/claudin across different treatment arms in participants from South Africa (n=40); (B) Ratio of CD1a/claudin across different treatment arms in participants from Uganda (n=40).
