# Supplementary material for: A randomized clinical trial of on-demand oral pre-exposure prophylaxis does not modulate lymphoid/myeloid HIV target cell density in the foreskin
Source: AIDS. 2023 Jun 6;37(11):1651–9. doi: 10.1097/QAD.0000000000003619 (PMC11175721; doi:10.1097/QAD.0000000000003619)
Supplement: Supplemental Digital Content [file aids-37-1651-s002.docx]

**Supplementary Table 1. Comparisons between trial arms for CD4+, CCR5+ cell density, distance of CD4+ cells from the epidermis and %CCR5 expression on CD4.** Results from mixed effects models (each image measurement is included in the model and clustering of measurements within each participant is accounted for by including a random effect). Geometric mean ratios are shown for all outcomes apart from percent CCR5 on CD4, as they were skewed.

|  |  | **CD4^+^/cm^2^** | |  | **CCR5^+^/cm^2^** | |  | **Distance from epidermis** | |  | **% CCR5 on CD4** | |
| --- | --- | --- | --- | --- | --- | --- | --- | --- | --- | --- | --- | --- |
| Reference Group | Comparator Group | Geometric mean Ratio (95% CI) | *P* |  | Geometric mean Ratio (95% CI) | *P* |  | Geometric mean Ratio (95% CI) | *P* |  | Geometric mean Ratio (95% CI) | *P* |
| **Effect of any PrEP versus no PrEP** | | | | | | | | | | | | |
| Control arm | Any PrEP | 0.92 (0.73, 1.18) | 0.53 |  | 0.91 (0.70, 1.17) | 0.44 |  | 1.04 (0.92, 1.18) | 0.51 |  | -2.9 (-10.6, 4.8) | 0.46 |
| **Overall effects of drug, dosage and interval** | | | | | | | | | | | | |
| F/TDF (all) | F/TAF (all) | 0.93 (0.77, 1.12) | 0.45 |  | 0.91 (0.75, 1.10) | 0.33 |  | 0.95 (0.87, 1.03) | 0.20 |  | -2.1 (-7.8, 3.6) | 0.46 |
| 2 tabs (both drugs) | 2+1 tabs (both drugs) | 0.91 (0.76, 1.10) | 0.35 |  | 0.89 (0.73, 1.08) | 0.23 |  | 1.02 (0.94, 1.11) | 0.58 |  | 0.7 (-5.0, 6.4) | 0.81 |
| 5 hours (all regimens) | 21 hours (all regimens) | 1.06 (0.88, 1.28) | 0.53 |  | 1.05 (0.86, 1.27) | 0.64 |  | 0.95 (0.87, 1.03) | 0.20 |  | 0.1 (-5.6, 5.8) | 0.97 |
| **Effects of dosage, separately for each drug** | | | | | | | | | | | | |
| F/TDF, 2 tabs | F/TDF, 2+1 tabs | 0.94 (0.72, 1.21) | 0.61 |  | 0.94 (0.71, 1.23) | 0.64 |  | 1.01 (0.91, 1.13) | 0.81 |  | 3.7 (-4.8, 12.2) | 0.39 |
| F/TAF, 2 tabs | F/TAF, 2+1 tabs | 0.90 (0.68, 1.19) | 0.44 |  | 0.84 (0.64, 1.11) | 0.23 |  | 1.04 (0.91, 1.18) | 0.60 |  | -2.3 (-10.0, 5.4) | 0.56 |
| **Effects of interval, separately for each drug and dosage** | | | | | | | | | | | | |
| F/TDF, 2 tabs (5h) | F/TDF, 2 tabs (21h) | 0.85 (0.59, 1.24) | 0.40 |  | 0.87 (0.58, 1.31) | 0.51 |  | 1.07 (0.92, 1.24) | 0.37 |  | -0.8 (-12.0, 10.3) | 0.89 |
| F/TDF, 2+1 tabs (5h) | F/TDF, 2+1 tabs (5h) | 1.12 (0.79, 1.60) | 0.53 |  | 1.12 (0.76, 1.65) | 0.56 |  | 0.88 (0.76, 1.01) | 0.08 |  | 5.1 (-8.0, 18.2) | 0.45 |
| F/TAF, 2 tabs (5h) | F/TAF, 2 tabs (21h) | 1.31 (0.83, 2.07) | 0.24 |  | 1.13 (0.72, 1.78) | 0.59 |  | 0.84 (0.68, 1.03) | 0.10 |  | -2.7 (-14.2, 8.9) | 0.65 |
| F/TAF, 2+1 tabs (5h) | F/TAF, 2+1 tabs (21h) | 1.02 (0.73, 1.43) | 0.89 |  | 1.09 (0.78, 1.52) | 0.61 |  | 1.02 (0.86, 1.20) | 0.84 |  | -1.3 (-12.0, 9.3) | 0.81 |
