# Supplementary material for: A randomized clinical trial of on-demand oral pre-exposure prophylaxis does not modulate lymphoid/myeloid HIV target cell density in the foreskin
Source: AIDS. 2023 Jun 6;37(11):1651–9. doi: 10.1097/QAD.0000000000003619 (PMC11175721; doi:10.1097/QAD.0000000000003619)
Supplement: Supplemental Digital Content [file aids-37-1651-s003.docx]

**Supplementary Table 3: Percentage of WLWH With Vitamin D Level Assessment in Pregnancy Per Year (2000-2018)**
